# Epigenetic Drift and the Generational Limit of Serial Somatic Cell Nuclear Transfer in Pigs

**DOI:** 10.3390/ani16101533

**Published:** 2026-05-17

**Authors:** Na Cheng, Muhammad Ameen Jamal, Helin Li, Mingjin Li, Qiue Xu, Hong-Jiang Wei, Wenmin Cheng

**Affiliations:** 1 Yunnan Key Laboratory of Porcine Gene Editing and Xenotransplantation, Yunnan Agricultural University, Kunming 650201, China; chengna2001@163.com (N.C.); drameen007@gmail.com (M.A.J.);; 2 Faculty of Animal Science and Technology, Yunnan Agricultural University, Kunming 650201, China; 3 College of Veterinary Medicine, Yunnan Agricultural University, Kunming 650201, China

**Keywords:** serial cloning, pig, epigenetics, reprogramming

## Abstract

Somatic cell nuclear transfer (SCNT) in pigs has been a widely used technique for producing gene-edited pigs for biomedical research, yet its wide-spread application through serial cloning remains markedly limited. Unlike in mice, where the serial cloning can be sustained across numerous generations, in pigs it is usually limited to only a few rounds. Specifically, porcine serial cloning has not been reported beyond three consecutive generations in live-born offspring, with blastocyst development rates declining from approximately 4.4% in G1 to 1–5% in G2–G3, and live-birth cloning efficiency (offspring/recipient) dropping sharply with each successive round. Compelling evidence suggests that cumulative epigenetic instability, incomplete embryo genome activation, DNA methylation reprogramming, persistent donor-cell memory, and imprinting disruption collectively erode transcriptional integrity across generations. Although several manipulations, including epigenetic modifiers, transiently improved the early development, they failed to sustain the reprogramming across several generations. Here, we synthesize comparative advances in serial cloning across species and propose that species-specific differences in chromatin plasticity and cytoplasmic reprogramming capacity define a porcine “reprogramming ceiling”. Deciphering and overcoming this barrier will be critical for advancing sustainable livestock engineering, xenotransplantation and translational medicine biotechnology.

## Simple Summary

This review summarizes the main barriers limiting serial cloning in pigs. Compared with mice, pigs show a marked decline in cloning efficiency over successive generations, which restricts the wider use of this technology. Shreds of evidence indicate that incomplete epigenetic reprogramming, including abnormal histone modifications, disrupted DNA methylation, and imprinting defects, is a major limiting factor. Existing treatments can only partly improve developmental outcomes. Emerging approaches, such as targeted epigenetic editing, single-cell multi-omics, and improved donor cell selection, may provide new ways to overcome these barriers. These advances could promote the future application of serial cloning in xenotransplantation, and regenerative medicine.

## 1. Introduction

The birth of the first cloned sheep, “Dolly,” demonstrated that somatic cell nuclear transfer (SCNT) technology can reprogram somatic cells to a totipotent state. To date, more than 20 mammalian species have been reprogrammed via SCNT, indicating its potential for reproduction of genetically valuable farm animals and rescuing endangered species [1]. Furthermore, the combination of SCNT and gene editing has been extensively utilized in producing gene-edited (GE) large animal models and xenotransplantation donors [2,3,4]. In addition to animal cloning, SCNT can also be applied to stem cell biology and human therapy [5]. Compared with mice, pigs are more similar to humans in anatomy and physiology and are therefore regarded as suitable large animal models for studying the mechanisms of complex diseases and as sources of organs for xenotransplantation. The lifespan and number of genetically edited positive cells are limited in the production of GE pigs [6], and culturing somatic cells for a long period increases the percentage of cells with chromosomal aberrations [7]. Therefore, increasing the effectiveness and cellular quality of donor cells for SCNT is crucial for producing GE donor pigs.

**Figure 1:**
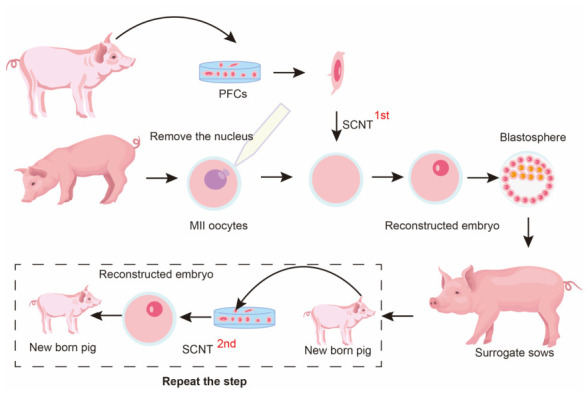
Serial cloning workflow. Schematic illustration of serial cloning in pigs. Somatic cells derived from cloned offspring are used as nuclear donors for subsequent rounds of SCNT across successive generations (G1–G3). This strategy enables genotype preservation and recloning, but repeated nuclear transfer may be associated with reduced developmental competence and cloning efficiency across generations. Figure created using Adobe Illustrator CC 2019, version 23.0.1, Adobe Inc., San Jose, CA, USA.

Serial somatic cell nuclear transfer, also known as multiple generation nuclear transfer, serial cloning, or recloning, is a technique in which the somatic cells from cloned individuals are used as a nuclear donor and transplanted into enucleated oocytes (Figure 1). Although serial cloning is not generally required for the routine production of transgenic or simple GE pigs, it can serve as a practical strategy in SCNT-based genetic engineering when complex GE pigs require multiple rounds of genetic modification, clonal screening, genotype validation, and expansion of confirmed donor lines [1,2]. GE pigs are generally produced by editing somatic cells in vitro, selecting correctly edited clones, and then using these verified cells as nuclear donors for SCNT. This is particularly relevant to xenotransplantation, where donor pigs often require combined genetic modifications targeting xenoantigen elimination, complement regulation, coagulation control, and immune compatibility [2,3,4]. However, transfection, clonal screening, and prolonged culture can reduce donor-cell quality, because donor-cell aging/senescence and culture-associated epigenetic abnormalities are important factors influencing SCNT efficiency and developmental competence [8,9]. Serial cloning can help mitigate this limitation: after a correctly modified cloned fetus or animal is obtained, newly derived fibroblasts or other somatic cells can be used as fresh donor cells for another round of SCNT [10]. Nevertheless, serial SCNT is not the only route for expanding GE livestock. Serial SCNT may be most useful as a short-term or specialized strategy for preserving and expanding confirmed complex genotypes [11], whereas natural breeding remains the preferred route for long-term population expansion when fertility and reproductive performance are normal [12,13]. In this way, serial cloning preserves the confirmed genotype while minimizing the need for continued manipulation and passaging of the original engineered cell line, thereby improving the practicality of producing genetically validated and complex multi-GE animals [10].

This advantage is particularly important to complex xenotransplantation donor lines, where recloning strategies have been applied for the generation or expansion of multi-GE pigs, including GTKO/hCD55/hCD59 triple-gene-modified pigs and pigs carrying multiple human complement- or coagulation-regulatory genes [11,14]. Nevertheless, serial cloning also requires a longer production cycle than single-round SCNT, and repeated nuclear transfer may allow epigenetic abnormalities to persist or accumulate across generations, thereby reducing developmental competence and cloning efficiency [13,15,16]. Therefore, clearly defining both the practical value and biological limitations of serial cloning is essential for improving its application in complex pig genetic engineering and for understanding the barriers that restrict sustained porcine serial SCNT.

Previously, we effectively utilized serial cloning technology to produce multi-GE pigs [11,17,18,19]. However, the cloning efficiency remains limited. Thus, we considered revisiting serial cloning in pigs, discussed the barriers hampering its success and proposed strategies for widespread use to provide some theoretical basis for future research.

## 2. Serial SCNT in Pigs: Progress, Limitations, and Comparative Context

Serial SCNT in pigs has progressed from early proof-of-concept experiments to the maintenance and refinement of increasingly sophisticated GE donor lines (Table 1). In 2007, Cho et al. reported one of the first clear demonstrations of serial pig cloning by producing first- (G1), second- (G2), and third-generation (G3) cloned piglets from transgenic fibroblasts carrying a truncated human thrombopoietin (hTPO) gene, thereby establishing that cloned pig lineages could be propagated across successive rounds of SCNT [15]. In 2008, this approach was further extended to α1,3-galactosyltransferase knockout (GTKO) pigs, showing that recloning could also support the expansion of gene-targeted pig lines relevant to xenotransplantation [20]. In the same year, Kurome et al. demonstrated that salivary gland progenitor cells could serve as donor nuclei for serial cloning, with pigs produced through at least the third generation without an obvious loss of production efficiency, supporting the biological feasibility of multigenerational recloning in pigs [12]. Subsequent studies in 2012 and 2013 confirmed the reproducibility of serial pig cloning across multiple generations using fibroblast-derived donor cells under different experimental settings [21,22]. More recently, the practical value of recloning has become even clearer in the context of complex pig genetic engineering. In 2020, GTKO/hCD55/hCD59 triple-gene-modified pigs were produced using serial cloning, highlighting its utility for producing xenotransplantation donor pigs carrying multiple engineered loci [11]. In 2021, GalT-null pigs expressing human complement- and coagulation-regulatory genes, including hCD55, hEPCR, and hTM were expanded via recloning, further illustrating its role in expanding pigs with clinically relevant xenoprotective modifications [14]. In 2022, OCT4-EGFP/SOX2-tdTomato reporter pigs were generated from fetal fibroblasts, indicating that the approach applies not only to xenotransplantation-related lines but also to functional reporter models [23]. Taken together, serial cloning is a valuable biotechnological tool for the maintenance, amplification, and refinement of GE pigs.

In contrast, serial cloning in other species has shown variable outcomes. In mice, serial SCNT has been explored far more extensively than in pigs. Wakayama and colleagues first achieved repeated recloning across multiple generations and later extended mouse serial cloning to 25 generations with trichostatin A-assisted protocols. A later study showed that serial cloning could be sustained up to the 58th generation, although cloning efficiency declined after the 27th generation and lethal structural mutations accumulated over time [24]. By comparison, serial cloning in cattle, goats, cats, and dogs has generally been reported only across two or three generations, often with reduced developmental or full-term efficiency in later rounds [16,25,26,27,28,29]. These cross-species findings suggest that, although serial cloning is biologically feasible in several mammals, its sustainability differs markedly among species. Overall, these findings indicate that serial cloning is feasible in pigs, but long-term sustainability remains more limited than in the mouse model.

**Table 1:** The progress in serial cloning of pigs and other species.

Species	Year	Donor Cell Type	Recipients/Oocytes (*n*)	Pups (*n*)	Generations	Cloning Efficiency	Study
Pig	2007	Newborn pig fibroblasts	11	5	G1	0.455	[15]
			37	4	G2	0.108	
			31	2	G3	0.065	
	2008	GTKO fetal fibroblasts	8	2	/	0.250	[20]
			7	6	/	0.857	
	2008	Salivary gland progenitor cells	2	2	G1	1.000	[12]
			3	13	G2	4.333	
			3	4	G3	1.333	
	2012	Fibroblast cells	/		G1	/	[21]
					G2		
					G3		
	2013	/	/		G1	4.40	[22]
					G2	3.50	
					G3	2.90	
	2020	Fibroblast cell	5	7	G1	1.400	[11]
			19	7	G2	0.368	
	2021	Fetal kidney cells/neonatal ear fibroblast	31	0	G1	0.000	[14]
			17	2	G2	0.118	
	2022	OCT4-EGFP/SOX2-tdTomato fetal fibroblasts	\		G1	\	[23]
					G2		
	2025	Fetal fibroblasts	4	1	G1	0.250	[3]
			17	28	G2	1.647	
	2025	Fetal fibroblasts	118	582 (Fetal)	G4	4.932	[2]
Mouse	2013	Cumulus cells	18	29	G1	1.611	[30]
			3	3	G2	1.000	
			4	3	G3	0.750	
			11	27	G25	2.455	
	2026	Cumulus cells	18	29	G1	1.611	[24]
			5	17	G26	3.400	
			12	34	G27	2.833	
Cattle	2004	Fibroblast cells	36	6	G1	0.167	[16]
			19	2	G2	0.105	
			30	0	G3	0.000	

Note: The cloning efficiency was calculated by offspring/recipients [31].

## 3. Epigenetic Drift Across Serial Generations

**Figure 2:**
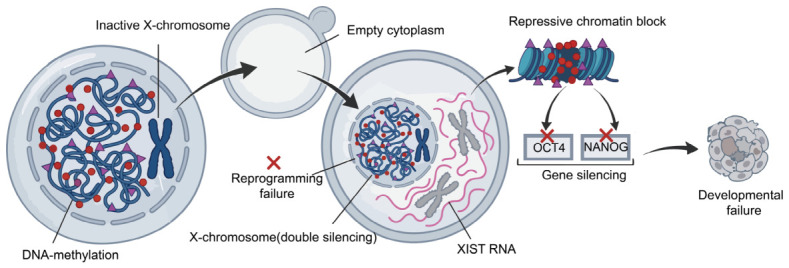
Epigenetic network disruption. Incomplete epigenetic resetting—including aberrant DNA methylation, repressive histone marks, imprinting instability, and X-chromosome dysregulation—impairs embryonic genome activation and drives cumulative failure of reprogramming. Figure created using Adobe Illustrator CC 2019, version 23.0.1, Adobe Inc., San Jose, CA, USA.

Epigenetic drift refers to the progressive accumulation of abnormal or unstable epigenetic states across successive rounds of cloning, even in the absence of changes to the underlying DNA sequence. In serial cloning, the donor nucleus must be reprogrammed again by the oocyte cytoplasm at each generation. Because this reprogramming is often incomplete, residual somatic epigenetic memory and newly introduced epigenetic errors may persist or accumulate across generations. Such defects can disrupt zygotic genome activation (ZGA), lineage specification, placental development, fetal growth, and postnatal viability, thereby compromising serial cloning efficiency. In pigs, the cloning efficiency decreased with successive cloning round, along with a decrease in the number of live and healthy offspring across generations [22]. This pattern suggests that repeated nuclear transfer imposes cumulative reprogramming stress on the donor genome. In pig cloning, incomplete erasure and re-establishment of epigenetic marks can affect several interconnected regulatory layers, including DNA methylation, histone modifications, X-chromosome regulation, and genomic imprinting (Figure 2). Because these mechanisms are essential for proper ZGA and lineage-specific gene expression, even subtle defects may be amplified during repeated cloning cycles.

Comparative studies in other livestock species support this interpretation and help explain why repeated recloning becomes increasingly difficult. In cattle, embryos derived from second-generation donor cells showed the poorest in vitro developmental competence and in vivo pregnancy outcomes, which have been associated with accumulated epigenetic abnormalities and increased DNA damage [16]. In goats, first-generation clones showed gene expression patterns closer to naturally bred controls, whereas second-generation clones displayed significant dysregulation of developmental genes such as *FGF10*, *MECP2*, and *GRB10*. Moreover, the differentially methylated region of *H19* was hypomethylated in both first- and second-generation clones relative to naturally conceived controls, indicating persistent imprinting abnormalities [27]. Altogether, these findings provide important comparative evidence that serial cloning can promote cumulative epigenetic disruption across generations. Among the various forms of epigenetic drift, abnormalities in DNA methylation, histone modification, and X-chromosome regulation appear to be especially important in determining the long-term sustainability of serial cloning.

### 3.1. DNA Methylation

In normal fertilized embryos, parental genome methylation undergoes a global erasure through active and passive mechanisms, followed by re-establishment around the implantation. However, this dynamic reprogramming process is often disrupted in SCNT embryos. Differentiated donor cells, such as fibroblasts, typically maintain high global DNA methylation levels. Following SCNT, this hypermethylated state may be aberrantly retained, resulting in the suppression of promoter regions of key pluripotency genes (*OCT4*, *NANOG*) that are required for EGA, thereby preventing their proper transcription [32]. During the early developmental stages of SCNT embryos (after the four-cell stage), an abnormal or premature wave of DNA “remethylation” occurs, contrasting with normal demethylation trajectory [33]. This premature and erroneous remethylation may further entrench an epigenetic state unfavorable for development, and contribute to subsequent developmental failure [33]. In pigs, this is particularly important because major ZGA occurs around the four-cell stage, meaning that defective methylation resetting at this stage can have disproportionate effects on subsequent development. In porcine SCNT embryos, the methylation status of multiple imprinting control regions (*H19*, *IGF2R*) is significantly altered, leading to dysregulated expression of imprinted genes [34]. These findings suggest that serial cloning does not fully reset the porcine donor epigenome but instead may permit the persistence or progressive drift of abnormal methylation marks across generations. This imbalance has been implicated in developmental abnormalities of cloned fetuses, such as large offspring syndrome and placental dysfunction. Development and gene expression data indicated that serial cloning does not enhance nuclear reprogramming of donor cells [13]. During serial cloning in pigs, alterations in DNA methylation patterns were identified; the methylation levels at *H19* DMR1 and DMR3 gradually decreased over three generations of cloning, while the *Igf2* DMR increased significantly in the G1 and remained elevated [35]. Similar dysregulations in growth factors (*FGF10*) and methyl-binding proteins (MECP2) have been documented in caprine serial SCNT, suggesting that the accumulation or “drift” of epigenetic errors acts as a major bottleneck for multi-generational viability [27]. Likewise, bovine serial cloning studies also support the view that cumulative epigenetic abnormalities contribute to declining developmental competence in later generations [16]. Consistent with this view, cumulative epigenetic abnormalities during serial cloning may compromise reprogramming of somatic cell nuclear transfer, and contributing to phenotypic abnormalities in the cloned animals [36]. Since DNA methylation controls imprinting stability, lineage-specific transcription, and promoter accessibility, its inadequate resetting offers a tenable molecular connection between donor-cell memory and developmental failure in serial cloning. Taken together, these findings support DNA methylation drift as a central mechanism contributing to the reduced developmental efficiency during serial cloning in pigs.

### 3.2. Histone Modification Barriers

Among the histone methylation markers studied, H3K9me3 and H3K27me3 have been identified as the major barriers to SCNT success [37,38]. In SCNT embryos, donor-cell-derived H3K9me3 can persistent abnormally on chromatin, particularly at telomeric and centromeric regions, as well as at key pluripotency gene loci, preventing the binding of reprogramming factors and gene activation. In pigs, persistent H3K9me3 is considered one of the major repressive chromatin barriers limiting efficient ZGA and developmental progression after SCNT. Demethylase Kdm4d expression can improve 3.4-fold blastocyst rates and the establishment of SCNT-derived embryonic stem cell cultures in mouse (26.0% ± 11.3% vs. 88.6% ± 3.9%) and human (4.2% vs. 26.8%) SCNT embryos [37,39]. Microinjection of KDM4 mRNA can also improve the developmental efficiency of pig SCNT embryos (57.8 ± 4.3% vs. 43.5 ± 1.8%), although this strategy is technically demanding, invasive, and time-consuming [40]. H3K9me3 is established by methyltransferases including SUV39H1, SUV39H2, and SETDB1. Specific inhibitor OTS186935 (targeting SUV39H2) and F5446 (targeting SUV39H1) can effectively correct aberrant H3K9me3 modification and ameliorate ZGA defects [41]. These findings indicate that targeted reduction in repressive histone methylation can partially overcome epigenetic resistance in porcine SCNT embryos. Consistent with this, serial SCNT of mouse was successfully achieved up to 25 generations by adding a histone deacetylase inhibitor, i.e., Trichostatin A (TSA) [30]. So, altering epigenetic modifications can increase the efficiency of serial cloning. However, targeted modification of histone methylation has not yet been explored in porcine serial cloning. Therefore, failure to erase donor-cell-derived repressive histone marks may represent a key mechanism through which chromatin memory persists across serial cloning generations.

SCNT embryos had lower histone acetylation levels compared to in vitro fertilized embryos [42], so increasing histone acetylation by inhibiting histone deacetylases can improve SCNT efficiency. TSA is commonly used as a histone deacetylase inhibitor (HDACi) and can improve the cloning efficiency in rhesus monkeys [43], pigs [44], rabbits [45], cattle [46], buffaloes [47], and cynomolgus monkeys [48] with optimal concentration and exposure time. In porcine SCNT embryos, representative HDACi-based interventions have produced clear improvements in blastocyst development: 50 nM TSA for 24 h increased blastocyst rates from 17.7% ± 4.9% to 46.4% ± 4.6% [44], 1 mM VPA (Valproic acid) for 14–16 h increased blastocyst development from 11.4% to 31.8% [49], and combined chaetocin plus TSA treatment improved blastocyst rates from 21.8% ± 1.1% to 35.4% ± 0.8% [50]. However, these improvements mainly reflect preimplantation development, and their ability to sustain full-term development or multigenerational serial cloning in pigs remains limited. In addition, TSA treatment can effectively reprogram the somatic cell nuclei and increase the success rate of mouse serial cloning [30]. However, TSA effects are species-, dose-, and endpoint-dependent, and the high concentration and long exposure time of TSA can produce toxicity to embryos. In cattle, several studies reported improved histone acetylation or preimplantation development after TSA treatment [51,52,53], whereas treatment of donor cells or cloned zygotes with chromatin-modifying agents did not necessarily improve full-term development. In sheep [54] and gaur–bovine interspecies SCNT [53], TSA-related benefits were also limited or inconsistent. Beyond TSA, several alternative HDACi have demonstrated improved reprogramming efficiency in porcine SCNT embryos. Notably, Scriptaid enhances blastocyst development with reduced toxicity compared to TSA [55]. Valproic acid improves embryonic competence, while sodium butyrate promotes chromatin accessibility and developmental progression [49,56]. Other HDACi such as oxamflatin, further support epigenetic remodeling [57]. These findings suggest that modulation of histone acetylation is not restricted to TSA, but represents a broader and potentially more flexible strategy for improving porcine nuclear reprogramming. Importantly, recent studies have reinforced the central role of HDACi in porcine nuclear reprogramming. Epigenetic modulation using HDACi significantly enhances chromatin accessibility and developmental competence in SCNT embryos [42,58], facilitates ZGA and transcriptional reprogramming [50,59], and shows synergistic effects when combined with histone methylation-targeting strategies. Thus, in pigs, histone modification barriers involve both persistent repressive methylation and insufficient acetylation, and both appear to contribute to the decline in developmental competence during repeated nuclear reprogramming. Taken together, current evidence suggests that the correction of histone modification abnormalities is a promising route to improve serial cloning outcomes in pigs, even though direct multigenerational pig studies targeting these marks are still limited.

### 3.3. X Chromosome Inactivation

In female mammals, dosage compensation is achieved through random inactivation of one X chromosome. The long non-coding RNA *Xist* gene can trigger X chromosome inactivation, but it is aberrantly expressed in animal SCNT. In the mouse, *Xist* was ectopically expressed from the active X chromosome in cloned mice of both sexes, and after inhibiting the aberrant expression of *Xist*, global gene expression was normal and the cloning efficiency was increased [36]. In pigs, *Xist* mRNA levels at the morula stage were aberrantly higher in SCNT embryos than in in vivo fertilized embryos. Injection of anti-*Xist* siRNA can significantly improve the birth rate of cloned healthy piglets [60]. These findings indicate that abnormal Xist activation is not merely a secondary epigenetic defect, but a major reprogramming barrier in porcine cloning. Therefore, defective X-chromosome regulation may represent an important contributor to cloning failure, particularly in female embryos. In pigs, this appears to be particularly important because aberrant Xist expression persists beyond the preimplantation stage and is associated with fetal abnormality. However, the ectopic *Xist* expression can be corrected autonomously after implantation in both embryonic and extraembryonic regions of cloned mouse embryos, but highly anomalous Xist expression persisted to the post-implantation stage in abnormal pig fetuses, which may be due to species-specific differences in *Xist* imprinting pattern [61]. Thus, compared with mice, pigs may be less able to correct abnormal X-chromosome inactivation after implantation, which could partly explain the greater developmental vulnerability of porcine cloned embryos and fetuses. Taken together, these observations suggest that defective X-chromosome regulation is an important component of epigenetic drift in serial pig cloning and may contribute to the decline in developmental competence across successive generations.

### 3.4. Zygotic Genome Activation

ZGA is the developmental transition during which control shifts from maternally stored RNAs and proteins to newly activated embryonic transcription. Dysregulation of ZGA is an important factor affecting the efficiency of SCNT. Activation of the embryonic genome in pigs occurs around the four-cell stage, a major transition point in porcine early embryonic development. Li et al. compared the global transcriptional dynamics during ZGA in SCNT embryos from five species: mouse, pig, cattle, goat, and sheep [62]. The results showed that pigs, mice, and bovines may share some overlapping but different sets of epigenetic challenges. SCNT embryos from mice and sheep exhibited insufficient activation of genes associated with pleuripotency and stem cell maintenance. SCNT embryos from pigs and sheep showed incomplete reprogramming of donor nuclei and led to a persistent fibroblast proliferation mode. Furthermore, pigs’ SCNT embryos exhibited widespread pathway overactivation and a trend of higher expression of nearly all chromosomes. These findings suggested that somatic cell nuclear reprogramming is a multifaceted and complex process, and both cytoplasmic and nuclear components of the oocytes should be taken into consideration simultaneously [62]. Importantly, the porcine pattern indicates that ZGA failure is not simply due to insufficient activation of embryonic genes, but also to incomplete silencing of the donor-cell transcriptional program, which may allow somatic memory to persist during early cleavage stages. This is particularly relevant in serial cloning because repeated rounds of SCNT may increase the likelihood that incomplete donor-nucleus reprogramming is carried forward across generations rather than fully erased at each cycle. Thus, although comparative analyses show that ZGA defects are a shared feature of SCNT embryos across species, pigs appear especially vulnerable to persistent donor-cell memory and chromosome-wide transcriptional dysregulation during the ZGA window.

## 4. Donor Cell Memory and Differentiation State

### 4.1. Type of Donor Cells

Donor cells provide the hereditary material necessary for embryonic development in serial cloning. A variety of donor cell types, including skin fibroblasts [13], fetal kidney cells [14], embryonic stem cells [63], and cumulus cells [30], can be successfully used as donor cells in serial cloning. However, the type of somatic cell can affect the efficiency of cloning. For instance, using cell lines derived from kidneys of cloned fetuses at 50 days of gestation as nuclear donors for serial cloning significantly increased blastocyst formation rates. Similarly, fetal or newborn piglet fibroblast cells successfully produced live third-generation cloned piglets [14,15]. The blastocyst development rate and cloning efficiency using transfected embryonic stem cells arrested at the M phase were significantly higher in the serial cloning group than that of the single cloning group (70% vs. 51%, *p* < 0.001) [63]. These results suggest that the developmental potential of cloned embryos is inversely correlated with the differentiation status of the donor cells, with less differentiated cells facilitating more efficient epigenetic reprogramming [64]. This may be due to the fact that donor cells from different tissues retain the epigenetic “memory” of their original cell types (such as incomplete DNA demethylation) after reprogramming, and this memory can affect the developmental potential of the reprogrammed cells. Thus, donor-cell effects on cloning efficiency are closely linked to the ease and completeness of epigenetic reprogramming. In pig and sheep SCNT embryos, the genes involved in fibroblast proliferation were significantly upregulated, which suggested incomplete reprogramming of donor nuclei and resulted in a persistent fibroblast proliferation mode during ZGA [62]. The cells with a higher degree of differentiation have more stable and complex epigenetic marks, such as DNA methylation and histone modifications, which are aimed at maintaining their specific cell identities.

The physiological state of donor cells can also influence serial cloning efficiency, particularly in terms of cell cycle synchronization and DNA integrity. Serum starvation markedly reduced the proportion of fibroblasts in the S-phase from 25.3% and 22.9% in non-starved controls to 5.4% and 3.9% in treated cells [65]. Reconstructed embryos derived from serum-starved fibroblasts exhibited slightly higher fusion rates and significantly improved development to the blastocyst (68.7% vs. 42.1%) and hatched blastocyst stages (55.3% vs. 28.6%) compared to non-starved cells [66]. However, Peura et al. thought serum starvation offers no advantages or disadvantages to cloning outcomes [67]. So, the outcome of cloning is still related to the cell line used or the result of other factors.

### 4.2. Telomeres

Telomeres are a repetitive DNA sequence, located at the ends of the chromosomes, and maintain chromosome integrity and genome stability in the cells. But telomeres shorten each time the cell replicates. For example, the terminal restriction fragment length in the first cloned sheep, Dolly, was smaller than that of age-matched control sheep [68]. However, some studies have reported the restoration of telomere length. In cloned piglets, the mean telomere length was elongated compared to nuclear donor fetal fibroblasts and age-matched normal piglets. In cloned cattle, the mean telomere length did not increase compared to the nuclear donor adult fibroblasts [69]. These results suggested that the cytoplast can reprogram the somatic cell nucleus and restore the telomere length to its totipotency stage. However, the deceased newborn SCNT calves had significantly shortened telomere lengths compared to newborn naturally conceived calves and newborn normal SCNT calves [70], which may be associated with abnormal development in the cloned calves.

There is still controversy over the restoration of telomere length in cloned animals. In mice, even after serial cloning for 25 generations, telomere shortening was not detected, and the genetic or epigenetic changes that hurt viability had not been introduced [30]. Tsai et al. used Terc^+/−^ TTFs (tail-tip fibroblasts) to generate G1 cloned mice, and G1-Terc^+/−^ ntESCs (derivative embryonic stem cells) were used in the second round of SCNT [71]. The results showed that telomere lengths of G1 ntESCs were elongated to a level comparable with that in wild-type ntESCs. Nevertheless, recloning did not further enable telomeres to elongate. In cattle, telomere length was restored to physiological levels in both G1 and G2 cloned calves, comparable to those of age-matched, naturally bred animals. Although telomeres were elongated relative to the donor cells in G2 clones, this increase remained within the species-specific physiological range [16]. Similarly, telomere length was maintained within normal physiological ranges across G1, G2, and G3 cloned offspring in pigs [12]. These results indicated that telomere shortening is unlikely to be the primary cause of serial cloning failure in pigs. Rather, telomere dynamics may act as an indicator of donor-cell history and reprogramming quality, whereas epigenetic instability appears to be a more consistent barrier to sustained serial SCNT.

## 5. Developmental and Transcriptional Dysregulation

**Figure 3:**
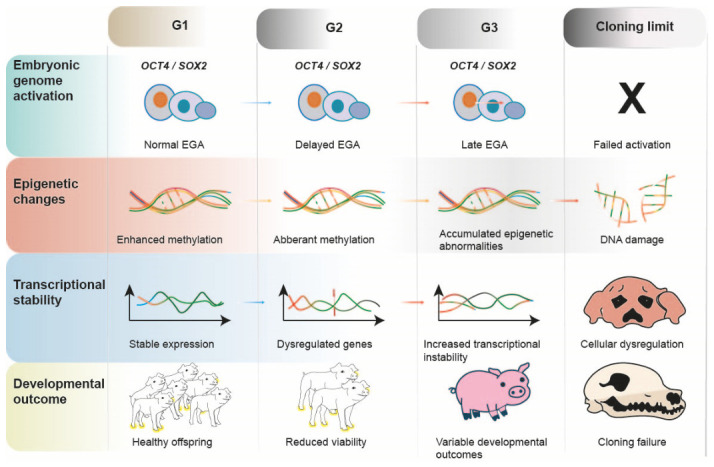
Progressive generational deterioration. Schematic model illustrating how repeated rounds of serial SCNT may lead to delayed EGA, aberrant DNA methylation, epigenetic drift, transcriptional dysregulation, reduced developmental competence, and eventual cloning failure. These cumulative abnormalities may define a species-specific “reprogramming ceiling” that limits long-term serial cloning efficiency, particularly in pigs and other large mammals. Figure created using Adobe Illustrator CC 2019, version 23.0.1, Adobe Inc., San Jose, CA, USA.

Pluripotent factors *OCT4* and *SOX2* of most reconstructed embryos were activated at the four-cell stage during first-round cloning, but their activation in most embryos was largely delayed until the morula stage in second-round cloning [23]. Phenotypic abnormalities have also been reported in some cloned offspring; for example, one cloned individual had a dimorphic facial appearance with severe hypertelorism and a broad prominent nasal bridge. Most studies report that serial cloning in pigs rarely exceeds the third generation, although viable and phenotypically normal offspring have been obtained in some G3 cases [12,15]. Supportive evidence from serial somatic cell chromatin transfer (SCCT), a related nuclear reprogramming approach, also indicates persistent transcriptional disruption. Transcriptomic comparison of IVF-derived blastocysts with first- and fourth-round SCCT blastocysts showed consistent upregulation of genes involved in cytoskeleton organization and cell-shape regulation, together with the downregulation of genes related to chromatin remodeling and stress responses in SCCT embryos [72]. Collectively, these molecular and transcriptional alterations converge into a generation-dependent decline in developmental competence (Figure 3).

## 6. Cytoplasmic Exposure and Serial Nuclear Transfer Attempts

In the traditional NT method, the donor nuclei are reprogrammed during exposure to the oocyte cytoplasm. Although the developmental pathways of oocytes in different species were conserved throughout evolution, there are still significant differences in the morphology and maturation of oocytes. Porcine oocytes are very dark and oocytes from bovine were dark due to the accumulation of lipid vesicles in the ooplasm [73], while oocytes from mice and women were translucent. And appropriate amount of lipid droplets (LDs) play an important role for successful embryonic development. LDs can efficiently exchange lipids and metabolites and interact with intracellular organelles [74]. Consistent with this, porcine IVM/ICSI studies indicate that cytoplasmic maturation and redox balance, particularly glutathione-related antioxidant capacity, are important determinants of embryo developmental competence [75]. However, whether LDs or redox status affect the cytoplasmic reprogramming capacity still requires further investigation.

In addition, in the process of NT, only a few reconstructed oocytes can obtain the complete capacity for full-term development. Extending the exposure time to the oocyte cytoplasm may increase reprogramming competence. In Wakayama’s experiment, cumulus cell nuclei were transferred into recipient oocytes. Twenty-four hours after this initial SCNT, pseudo-MII spindles (pMIIs) derived from these reconstructed oocytes were extracted and injected into newly collected, enucleated oocytes. But there were no significant differences in cloning efficiency across three rounds of serial SCNT: 2.02% (fresh), 2.43% (6 h exposure), and 2.25% (24 h exposure) [25]. Ono et al. reported that fetal fibroblasts arrested at metaphase were transferred to enucleated MII oocytes, and after activation, the resultant MII apparatus was transferred again to enucleated fertilized 1-cell embryos [76]. The single transfer technique failed to yield any healthy offspring. On the contrary, serial transfer yielded 4 live pups from 20 recipients in G1 (0.2) and 1 live pup from 18 recipients in G2 (0.06). Therefore, early transcripts in the cytoplasmic environment of fertilized embryos can support later development, or donor cell-derived products of cloned embryos are diluted by the second nuclear transfer, and their ovicidal effect is reduced.

In addition, the effect of hand-made cloning (HMC) and serial hand-made cloning (SHMC) was compared [77]. The cloning efficiency for SHMC and HMC was 0.06 (1 pup/16 recipients) and 0.04 (1 pup/23 recipients), respectively, and cloned calves from both groups that reached term were healthy postnatally and displayed normal viability. However, one serial nuclear transfer preterm fetus showed renal and hepatic abnormalities. The reason for this was that the reprogramming of donor cell nuclei may still be a critical issue. Overall, these data indicate that cytoplasmic exposure is required but not sufficient for full nuclear reprogramming; the timing, quality, and molecular composition of the recipient cytoplasm may be more significant than exposure time.

## 7. Fetal Loss

In commercial pig farming, spontaneous fetal loss remains a significant concern. Approximately 20–30% of genetically normal pigs are lost during the peri-implantation period (day 12–30 of pregnancy) [78,79,80] and 10–15% of these are lost at mid-to-late gestation (day 50–90 of the 114-day porcine gestation length) [81,82]. Although this pattern has also been documented in other livestock, its impact is especially relevant in pigs because high fetal loss substantially limits the practical efficiency of cloning and serial cloning. Thus, evaluation of serial cloning in pigs should include not only cleavage or blastocyst development, but also implantation success, fetal maintenance, and live birth. In SCNT, more than 90% cloned embryos fail to survive to term and are aborted at different gestational [83], similarly SCNT embryos displayed a high rate of embryonic mortality before day 14 in bovine [84]. Epigenetic errors can occur at either of the two stages: the first stage is pre-implantation, involving the erasure of epigenetic patterns in the terminally differentiated somatic cells and the reestablishment of a totipotent embryonic epigenetic state; the second stage is post-implantation development, involving redifferentiation of the totipotent embryonic status to various differentiated somatic cell types. These epigenetic abnormality may be responsible for SCNT-related miscarriages [83]. This finding is particularly important for pigs because it indicates that post-implantation fetal loss is not merely a nonspecific consequence of poor embryo quality, but can arise from a defined epigenetic defect affecting placental and fetal maintenance. Yu et al. showed aberrant silencing of RTL1 was the common epigenetic reason for pregnancy failure and RTL1 restoration can rescue the fetal loss of cloned pig [83]. Therefore, the failure of pregnancy after transplantation should also be a key area of concern. In the context of serial cloning, this issue may become even more important, because incomplete epigenetic reprogramming accumulated across successive cloning rounds could further increase the risk of implantation failure, placental dysfunction, and fetal loss. Moreover, the assessment of serial cloning success in pigs requires monitoring full-term outcomes and not merely early embryo. Overall, fetal loss should be regarded as a central biological and practical endpoint in porcine serial cloning, rather than simply a downstream consequence of low embryo production efficiency.

## 8. Why Epigenetic Interventions Are More Durable in Mice than in Pigs

In mice, serial cloning has shown a remarkable ability to apply epigenetic treatments to sustain multi-generational reprogramming. Notably, TSA-treated mice were able to successfully extend serial SCNT to 58 generations [24]. This finding indicates that pharmacological relaxation of chromatin can sustain cloning competence across multiple rounds of nuclear transfer. TSA-mediated histone acetylation increased chromatin accessibility and enabled the reactivation of embryonic transcriptional pathways. Additionally, it has been shown that removing restrictive histone methylation markers improves cloning results. Overexpression of the demethylase *Kdm4d* can alleviate H3K9me3, which has been identified as a major epigenetic barrier to SCNT success [37,38]. This leads to a 3.4-fold increase in blastocyst formation and improved establishment of SCNT-derived embryonic stem cell cultures in mouse and human embryos [37,39]. Global gene expression patterns were restored and cloning efficiency was much enhanced in mouse cloned embryos when aberrant *Xist* expression was suppressed by siRNA [36]. Together, these findings suggest that in mice, several major epigenetic barriers are sufficiently reversible that targeted intervention can produce durable functional improvement. All of these results point to the possibility of restoring reprogramming capacity in the murine system through targeted modification of epigenetic barriers.

Conversely, comparable treatments in pigs have shown more limited and less durable results. KDM4-based strategies have improved SCNT reprogramming efficiency, including in porcine embryos, although their application remains invasive and technically demanding [37,39,40]. Rhesus monkeys, pigs, rabbits, cattle, buffaloes, and cynomolgus monkeys are among the species in which TSA therapy has increased cloning efficiency [43,49,52,53,54]. Nevertheless, the positive effects are greatly dependent on concentration and exposure length, and excessive treatment causes embryotoxicity. In pigs, these interventions often improve preimplantation development, but their benefits are usually less stable across the full developmental trajectory. Interspecies diversity in epigenetic responsiveness is also shown by certain investigations that found no discernible benefit after TSA treatment in cloned cattle [51,52,85,86], sheep [54], and gaur–bovine interspecies SCNT [53]. In pigs, anti-*Xist* siRNA injection raised the birth rate of cloned piglets [60], but aberrant *Xist* expression continued into the post-implantation stage in aberrant fetuses [61], indicating unstable or insufficient repair of X-chromosome inactivation. This suggests that, in pigs, the correction of one molecular defect may improve one developmental window without fully restoring the broader epigenetic architecture required for normal fetal development.

All of these findings suggest that although epigenetic treatments can temporarily correct certain molecular flaws, they are unable to completely stop the accumulation of reprogramming disorders in pigs over successive generations. A likely reason is that epigenetic dysregulation in pigs is broader and more interconnected, involving histone methylation, histone acetylation, X-chromosome regulation, zygotic genome activation, imprinting stability, and post-implantation fetal maintenance. The disparity between the porcine generational ceiling and the much longer serial cloning continuity observed in mice [30] points to species-specific variations in chromatin architecture, epigenetic plasticity, and the durability of reprogramming correction. Therefore, treatments that are effective in overcoming epigenetic barriers in mice may be insufficient to reverse the cumulative drift observed during repeated serial cloning of pigs. Thus, the key difference is not that epigenetic interventions work in mice but fail in pigs; rather, these interventions appear to rescue mouse embryos more completely and more durably, whereas in pigs they usually provide only partial and transient improvement.

## 9. Breaking the Reprogramming Ceiling

The limited durability of current epigenetic interventions indicates that future strategies should move beyond broad chromatin modulation toward more precise and mechanism-driven correction [37,39,40,41]. In pigs, improving serial cloning efficiency will likely require coordinated regulation of several key barriers, including repressive histone marks [37,38,39,40,41], imprinting instability [35], X-chromosome dysregulation [60], donor-cell memory [62], and insufficient cytoplasmic reprogramming capacity [87]. Therefore, the next stage of research should focus on identifying generation-specific reprogramming defects and developing targeted interventions that can stabilize embryonic transcriptional programs across successive rounds of SCNT [72].

Given the observed changes in imprinting regulatory regions such as *H19* and *IGF2* during porcine serial SCNT [35], together with the dysregulated expression of genes such as FGF10 and MECP2 in caprine serial SCNT [27], a more focused approach to correcting locus-specific epigenetic defects may be more effective. Targeted correction of crucial regulatory regions may reduce unintended transcriptional disruption while preserving developmental gene networks, as opposed to altering chromatin states globally.

Another important component for improvement is X chromosome control. Sustained Xist dysregulation in aberrant pig fetuses [61] indicates that temporary correction might not be enough, even if suppression of aberrant Xist expression enhances cloning efficiency in mice [36] and raised the birth rate of cloned piglets after anti-*Xist* siRNA injection [60]. To avoid transcriptional imbalance during serial cloning, dosage-compensation mechanisms may need to be stabilized during development.

Furthermore, prolonged exposure of donor nuclei to the oocyte cytoplasm does not considerably increase cloning efficiency across rounds, according to serial nuclear transfer tests [87]. Serial transfer into the fertilized cytoplasm produced slight improvements over single transfer [76], but these advantages did not result in long-term success across generations. This finding suggests that pigs may have an innately limited potential for cytoplasmic reprogramming, and that, to facilitate full epigenetic resetting, it may be necessary to increase maternal factor availability or remodeling activity.

Finally, comparative transcriptome investigations of SCNT-derived embryos demonstrated significant gene dysregulation in chromatin remodeling, cytoskeletal architecture, and stress responses [72]. These changes are probably the result of generational progressive epigenetic drift. Therefore, systematic multi-generational assessment of transcriptional and epigenetic states may help identify instability trajectories early and inform more logical intervention approaches. To provide a clearer overview of the major molecular interventions that may improve porcine SCNT and serial cloning efficiency, the main tools and strategies reported in previous studies are summarized in Table 2.

## 10. Porcine Serial Cloning Reprogramming Ceiling Model

**Figure 4:**
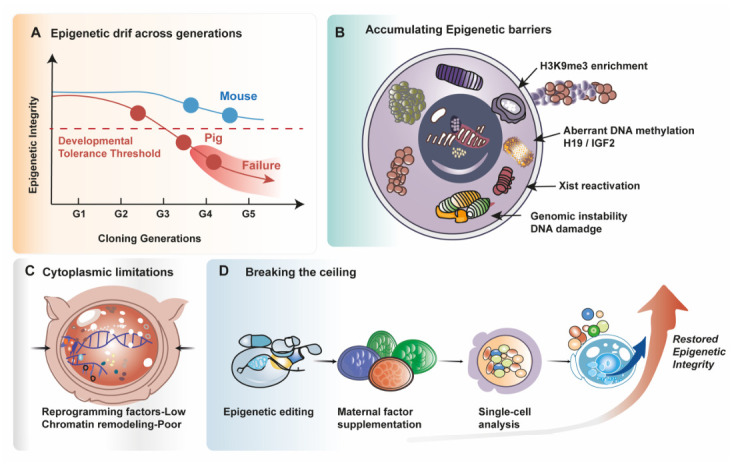
Porcine reprogramming ceiling model. (**A**) Serial cloning in pigs exhibits progressive decline in efficiency across generations, culminating in an early generational limit. (**B**) Persistent donor-derived epigenetic marks, including aberrant DNA methylation, repressive histone modifications, imprinting instability, and Xist dysregulation, accumulate across rounds. (**C**) Limited cytoplasmic reprogramming capacity fails to fully erase accumulated epigenetic memory. (**D**) The interaction between nuclear epigenetic resistance and restricted cytoplasmic remodeling establishes a species-specific developmental threshold: the porcine reprogramming ceiling. Figure created using Adobe Illustrator CC 2019, version 23.0.1, Adobe Inc., San Jose, CA, USA.

Based on the preceding evidence, we suggest a porcine serial cloning reprogramming ceiling model (Figure 4A). Several porcine studies report declining efficiency across successive recloning rounds, supporting a progressive loss of developmental competence [12,15,22]. Serial cloning in pigs is characterized by a persistent loss of developmental competence across generations, ultimately reaching a generational limit at the third round [12,15]. Porcine serial cloning fails to attain sustained multi-generational stability, demonstrating the presence of species-specific restrictions, in contrast to mice. The ceiling results from the accumulation of epigenetic defects across several cloning rounds, as seen in Figure 4B. Complete nuclear reprogramming is hampered by persistent donor-derived DNA methylation patterns, especially at imprinting regulatory sites like *H19* and *IGF2* [35], repressive histone modifications like H3K9me3 [37,38], and abnormal *Xist* expression [60,61]. Individual treatments aimed at these obstacles can temporarily enhance developmental outcomes [37,39,60], but they are unable to completely restore epigenetic integrity across generations.

In addition to nuclear epigenetic resistance, repeated exposure of donor nuclei to the oocyte cytoplasm has no substantial effect on cloning efficiency [87], and serial transfer into fertilized cytoplasm offers very minor improvement [76]. These results imply that accumulated epigenetic memory during serial cloning may not be completely erased by cytoplasmic reprogramming ability (Figure 4C). The interplay between enduring nuclear epigenetic barriers and limited cytoplasmic remodeling capacity thus creates a developmental threshold that prevents additional cloning cycles.

Together, these models conceive the failure of porcine serial cloning as the result of cumulative and interacting reprogramming barriers rather than a single molecular flaw. Coordinated correction of locus-specific epigenetic aberrations, stabilization of transcriptional control, and reinforcement of cytoplasmic reprogramming capacity over subsequent generations would probably be necessary to overcome this ceiling (Figure 4D).

## 11. Conclusions and Future Perspectives

Serial cloning represents a transformative tool for the large-scale propagation of elite germplasm and the generation of complex GE animal models. However, its multi-generational application in large mammals, particularly pigs, remains markedly more limited than that in mouse models. This interspecies disparity likely mirrors intrinsic differences in epigenetic plasticity and the narrowed window for EGA in large domestic animals, which in turn restricts the effective clearance of donor cell epigenetic memory. Accumulating evidence highlights that the progressive epigenetic aberrations, notably recalcitrant heterochromatin marked by H3K9me3 and destabilized imprinting control regions like *H19/IGF2*, represent a primary barrier to sustained developmental competence across successive cloning generations. Although chemical modulators like HDAC inhibitors have offered marginal improvements, they fail to rectify deep-seated, site-specific epigenetic defects. Further progress will probably rely on the transition from global epigenetic modulation to precision epigenetic corrections. Harnessing CRISPR-dCas9-mediated targeted methylation or acetylation to “repair” specific reprogramming-resistant regions offers a promising pathway to overcome generational stagnation. Along with the integration of single-cell multi-omics, it will facilitate the identification of novel porcine-specific reprogramming factors, potentially allowing for the artificial creation of a more permissive cytoplasmic environment. Future strategies should also explore the utilization of extended pluripotent stem cells as donor nuclei to leverage their superior chromatin accessibility, ultimately realizing the full potential of this technology in sustainable animal biotechnology and regenerative medicine. Altogether, these developments could lay down the foundation for overcoming present generational barriers and realizing serial cloning’s full potential in regenerative medicine and sustainable animal biotechnology, thereby providing a theoretical basis for future research.

## Figures and Tables

**Table 2:** Summary of molecular tools and strategies for improving porcine SCNT and serial cloning efficiency.

Category	Representative Examples	Potential Role in Serial Cloning	References
HDAC inhibition	TSA, Scriptaid, VPA, sodium butyrate, oxamflatin	Promotes chromatin relaxation and embryonic gene activation	[30,42,44,50,55,57]
Histone demethylation	KDM4A/KDM4D overexpression, SUV39H1/SUV39H2 inhibition	Reduces repressive chromatin barriers and facilitates nuclear reprogramming	[37,38,40,41]
Xist suppression	Anti-Xist siRNA	Improves dosage compensation and cloned piglet birth rate	[60,61]
DNA methylation modulation	Regulation of abnormal remethylation and H19/IGF2-related imprinting regions	Supports epigenetic resetting and may reduce imprinting-related developmental abnormalities	[33,34,35]
Locus-specific epigenetic correction	Targeted correction of methylation, acetylation, or repressive histone marks	Provides a more precise strategy than global epigenetic modulation	[35,37,40,41]
Donor cell optimization	Fresh or less differentiated donor cells, cell-cycle synchronization, reduced passage number	Improves donor-cell quality and reduces reprogramming burden before SCNT	[10,15,63,64,65,66,67]
Multi-omics-guided screening	Single-cell RNA-seq, ATAC-seq, DNA methylome analysis, multi-omics integration	Identifies generation-specific defects and informs mechanism-based intervention strategies	[62,72]

## Data Availability

No new data was created or analyzed in this study. Data sharing is not applicable to this article.
